# Study of age-related changes in postural control during quiet standing through Linear Discriminant Analysis

**DOI:** 10.1186/1475-925X-8-35

**Published:** 2009-11-18

**Authors:** Guilherme L Cavalheiro, Maria Fernanda S Almeida, Adriano A Pereira, Adriano O Andrade

**Affiliations:** 1 Biomedical Engineering Laboratory, Faculty of Electrical Engineering, Federal University of Uberlândia, Campus Santa Mônica, Bloco 1E, Av. João Naves de Ávila, 2121, Uberlândia, Minas Gerais, 38.408-100, Brazil

## Abstract

**Background:**

The human body adopts a number of strategies to maintain an upright position. The analysis of the human balance allows for the understanding and identification of such strategies. The displacement of the centre of pressure (COP) is a measure that has been successfully employed in studies regarding the postural control. Most of these investigations are related to the analysis of individuals suffering from neuromuscular disorders. Recent studies have shown that the elderly population is growing very fast in many countries all over the world, and therefore, researches that try to understand changes in this group are required. In this context, this study proposes the analysis of the postural control, measured by the displacement of the COP, in groups of young and elderly adults.

**Methods:**

In total 59 subjects participated of this study. They were divided into seven groups according to their age. The displacement of the COP was collected for each subject standing on a force plate. Two experimental conditions, of 30 seconds each, were investigated: opened eyes and closed eyes. Traditional and recent digital signal processing tools were employed for feature computation from the displacement of the COP. Statistical analyses were carried out in order to identify significant differences between the features computed from the distinct groups that could allow for their discrimination.

**Results:**

Our results showed that Linear Discrimination Analysis (LDA), which is one of the most popular feature extraction and classifier design techniques, could be successfully employed as a linear transformation, based on the linear combination of standard features for COP analysis, capable of estimating a unique feature, so-called *LDA-value*, from which it was possible to discriminate the investigated groups and show a high correlation between this feature and age.

**Conclusion:**

These results show that the analysis of features computed from the displacement of the COP are of great importance in studies trying to understand the ageing process. In particular, the *LDA-value *showed to be an adequate feature for assessment of changes in the postural control which can be related to functional changes that occur over the ageing.

## Background

Human beings adopt a number of strategies to maintain its body balance in the upright position. This is an extremely difficult task that demands a complex system, which is responsible to keep the projection of the centre of gravity of the subject over the base-of-support.

The centre of gravity tends to unbalance the person, who through the visual, somatosensorial and vestibular systems, perceives such disequilibrium and sends signals for the nervous system, that in turn acts on the muscles to modify the position (centre of pressure - COP) and the intensity of the reaction force of the ground under the plant of the feet, being compensated the disequilibrium and making it possible the complicated task to remain in the upright position. As the displacement of the COP reflects the behavior of the corporal segments to maintain itself in balance, this signal has been widely used to study the postural control [1-26].

As part of the ageing process a number of changes occur in the postural control. Some of them are reflected on the displacement of the COP. For this reason it is possible to find a great number of studies that compare the postural control of young and elderly adults [1,2,4-7,9,10,12-14,16,17,19-23,25,26]. Very often they employ a force plate to measure the displacement of the COP, resulting in a signal in the Antero-Posterior axis (AP) and another in the Medium-Lateral axis (ML). The resulting trajectory (RD) of these two axes may be used as a complementary information in the analysis [1,4-7,12,21].

Several types of methods are used to analyze the displacement of the COP. Some of them employ traditional tools, such as the *total displacement*, *mean velocity*, *RMS value*, *mean frequency *and the *confidence ellipse area *[13,14,16,19,21]. Others use mathematical techniques from statistical mechanics, assuming that the displacement of the COP is a random process [1,2,4-7,9,10,17,19,20,22], for instance, the *Stabilogram Diffusion Analysis *(SDA), *Detrended Fluctuation Analysis *(DFA) and *Analysis R/S*.

Although it is possible to find a number of studies in this area, there is a lack of investigations that seek features computed from the displacement of the COP that may reflect changes in the postural control over the ageing. In this context this study investigates how traditional and recent tools for feature estimate can be employed to investigate the correlation of changes in the displacement of the COP over the ageing.

## Methods

In total 59 healthy subjects (i.e., without clinical evidence or history of suffering from any neuromuscular disorder, as assessed by a seasoned neurologist) participated in the experiments.

All subjects gave their informed consent prior to participation in the study, which was approved by the Ethical Committee of the Federal University of Uberlândia - Brazil.

The subjects were classified into the following groups according to their ages, where N is the number of subjects within the group:

• Group 1 (N = 10; 20 to 29 years old);

• Group 2 (N = 10; 30 to 39 years old);

• Group 3 (N = 8; 40 to 49 years old);

• Group 4 (N = 10; 50 to 59 years old);

• Group 5 (N = 9; 60 to 69 years old);

• Group 6 (N = 8; 70 to 79 years old);

• Group 7 (N = 4; 80 to 89 years old).

The displacement of the COP was recorded by a commercial force plate (BioDynamicsBr model of the DataHominis company). The sampling frequency was set to 150 Hz. The collected signal was filtered by using a low-pass filter with cutoff frequency of 30 Hz.

During the recordings, the subject remained for 30 seconds on the force plate in the upright position, with the arms on the laterals of the body, with the feet forming an angle of 20 degrees and the heels moved away from 2 cm. This procedure was performed 3 times for each condition (i.e., opened eyes and closed eyes). Each subject was asked to minimize the postural sway and, during the opened-eye condition, to stare at a fixed point at 2 m from his eyes.

In order to compute features from the displacement of the COP traditional tools (*mean velocity, total displacement, RMS value, range, frequency domain features *and *Confidence Ellipse*) and mathematical techniques from statistical mechanics (*DFA*, *SDA*, *Analysis R/S *and *Approximate Entropy*) were employed. These techniques are described in the next section.

Each feature computation technique was applied for the signals of the two axes, ML and AP, and for the two conditions, i.e., opened eyes and closed eyes.

For each subject, it was calculated the mean value of each feature, obtained from the three repetitions for each experimental condition (CE, OE).

For each feature, it was applied the analysis of variance (ANOVA) in order to verify whether there exist statistical significant differences between the young (formed by groups 1 and 2) and elderly groups (formed by groups 6 and 7). These groups were defined based on previous studies carried out in the area [1,2,4-7,9,10,12-14,16,17,19-23,25,26]. A probability value (*p-value*) less than 0.05 was an indicative of significant differences between the two groups.

In order to investigate a possible correlation between age and the computed features we estimated the Pearson's correlation coefficient (*r*) and the correspondent p-value. Note that we took into account all subjects from groups of 1 to 7 in this analysis. Furthermore, we also studied possible correlation between distinct features. When we obtained *r *> 0.9 we considered a strong correlation between the variables.

As no computed feature from the displacement of the COP yielded a discrimination of the seven groups, the LDA technique was used to estimate a single feature, so-called *LDA-value*, which was a combination of all computed features from the displacement of the COP. The correlation between the *LDA-value *with age through the Pearson's correlation coefficient, and the potential of the *LDA-value *as a discriminative feature capable of characterizing groups from 1 to 7 was investigated.

### Description of the features

For all features described in this section, *d*_*cop *_is the signal of the displacement of the COP, *N *is the total number of samples and *T *is the sampling period.

### Mean velocity

The *mean velocity *(*MV*) of the displacement of the COP (*d*_*cop*_) is given by (2), where *v*(*n*) is the instantaneous velocity given by (1).(1)

### Total displacement

The *total displacement *(*TD*) of the displacement of the COP is calculated by summing up all the distances of two consecutive samples, as shown in (3).(3)

### Root mean square

The *root mean square *(RMS) is a statistical measure of the magnitude of a varying quantity, and it is calculated from (4).(4)

### Range

*Range *is a quantity defined as the difference between the maximum value (maximum global) and the minimum value (minimum global) of the signal. It is computed as shown in (5).(5)

### Frequency domain features

The *frequency domain features *were obtained from the power spectrum, *S*_*x*_, of the signal, which was estimated through the Fourier Transform and *f *is the frequency vector of the *S*_*x*_.

The *total power *(*P*_total_) given in (6), *mean frequency *(*f*_mean_) given in (7), *peak frequency *(i.e., the frequency where *S*_*x *_is maximum), *F50 *(also known as the *median frequency *of the signal, where 50% of *total power *of the signal is below *F50*) and *F80 *(where 80% of *total power *of the signal is below *F80*) were estimated.(6)

### Confidence ellipse

The *95% confidence ellipse area *is a method to estimate the confidence area of the COP path on the force plate that encloses approximately 95% of the points on the COP path [19,21]. The procedure to calculate the 95% confidence ellipse area is shown from Equation (8) to (12), where *S*_*AP *_and *S*_*ML *_are the standard deviation of the displacement of the COP on AP and ML axes and *S*_*APML *_is the covariance between the displacement of the COP in the AP and ML axes.(8)

### Detrended fluctuation analysis (DFA)

DFA is a tool for analysis of random signals that estimates the *α *exponent which may characterize the nature of the time-series [1,9,19].

The time series *d*_*cop *_is divided into intervals of *τ *samples without overlapping. For each interval, the mean value  (13), the function *y(n) *(14) and the linear model *z(n) *(15) are calculated, where *a *and *b *are the angular and linear coefficients of the linear model for this interval, and *n *is the current sample.(13)

The fluctuation function *FF(k) *for each interval *k *is calculated by (16), where *1 *≤ *k *≤ *N/τ*. Then the mean value of *FF(k) *for all intervals is estimated as in (17).(16)

A behavior *F(τ) *~ *τ*^*α *^is expected, where the characteristic exponent *(α) *can be extracted through the inclination of the straight line in the graph *log(F(τ)) vs log(τ)*.

An exponent *(α) *lesser than 0.5 characterizes an anti-persistent signal; α greater than 0.5 characterizes a persistent signal; and a white noise has a exponent *(α) *equal to 0.5.

### Stabilogram diffusion analysis (SDA)

This method, which is based on the work of Collins and DeLuca, 1993 [4], relates the displacement of the COP (*d*_*cop*_) to a random walk motion. For this, the calculation of the amplitude distances between successive samples, separated for a given time interval (represented by *m *samples) is carried out, and then, the average of these distances is calculated, as shown in (18), where *m *is an integer number that corresponds to a time interval between any two samples.(18)

The graph *vs *Δ*t *generally shows two distinct linear regions (*short-term *and *long-term*), and each region is characterized by: a diffusion coefficient (*D*), that can be obtained from the graph through the expression  = 2*D*Δ*t*; a scaling exponent (*H*), obtained through the expression  ∝ Δ*t*^2*H*^; and a critical point that divides the graph into two regions (the long and short term).

### Analysis R/S (Hurst exponent)

This analysis was defined by Hurst [9,19] to detect the "persistence" or long- term memory in time series. The procedure is described below.

The time series *d*_*cop *_is divided into intervals of *τ *samples. For each interval, the function *y *is calculated by (19), where *n *is the current sample and  is the mean of *d*_*cop *_in the current interval.(19)

The next step is to calculate the function *R(τ) *given by (20) and the standard deviation S(*τ*) given by (21) of this interval.(20)

Thus, for each value of *τ*, the value of *R(τ)/S(τ) *is calculated. It is expected the behavior *R(τ)/S(τ) *~ *τ*^*Hr*/*s*^, where the exponent *H*_*R*/*S *_can be extracted through the inclination of the straight line on the graph *log(R/S) vs. log(τ)*. In general the exponent can vary from 0 to 1. If 0.5 <*H*_*R*/*S *_≤ 1, the time series is persistent, with effects of long term memory. If *H*_*R*/*S *_< 0.5, the time series is anti-persistent, and a white noise is represented by *H*_*R*/*S *_= 0.5.

### Approximate entropy

*Approximate entropy *(ApEn) is a tool used to quantify the regularity of a signal [27], returning a value between 0 and 2, where 0 represent a predictable signal through its previous samples, like a sinusoidal signal, and a value close to 2 represents an unpredictable signal, such as a white noise.

In order to calculate the approximate entropy of the time series *d*_*cop *_is necessary to select values for the parameters *m *(length of a pattern) and *r *(criterion of similarity or tolerance of comparison). If a signal window of *m *samples beginning at sample *i *is denoted by *p*_*m*_(*i*), then two patterns *p*_*m*_(*i*) and *p*_*m*_(*j*) will be similar if the difference between any pair of corresponding measures of the patterns is less than *r*, therefore [*d*_*cop*_*(i+k) *- *d*_*cop*_*(j+k)*]<*r*, for 0 ≤ k < m.

Being *P*_*m *_the set of all patterns of length *m *in *d*_*cop*_, the fraction of patterns of length *m *that resembles the pattern of the same length starting at *i *is *C*_*im*_(*r*). C_*im*_(*r*) is the number of patterns in *P*_*m *_that are similar to *p*_*m*_(i). In this case, *C*_*im*_(*r*) can be calculated for each pattern in *P*_*m*_, setting up *C*_*m*_(*r*) as the average of these values. *C*_*m*_(*r*) measures the regularity or the frequency of similar patterns to a certain pattern in *d*_*cop *_with a window length equal to *m*, obeying the tolerance *r*. Therefore, the approximate entropy of *d*_*cop *_can be defined as in (22).(22)

The approximate entropy (ApEn) measures the similarity between patterns with lengths *m *and *m+1*. This technique was applied to the displacement of the COP with a value of *m *(window length) equal to 2 and *r *(tolerance) equal to *0.2SD(d*_*cop*_), where *0.2SD(d*_*cop*_) is the standard deviation of *d*_*cop*_, as suggested by Pincus [27].

### Linear Discriminant Analysis (LDA)

The LDA is a known method for data classification and dimensional reduction. Through this method it is possible to project a multidimensional data set in only one dimension, resulting in a single feature [28-30].

In this study, we used the LDA to verify whether the combination of the computed features from the displacement of the COP could discriminate the seven groups in analysis. For each subject a pattern vector was created by grouping all computed features from the displacement of the COP, and the LDA was applied for dimension reduction. The pattern vector, *v*_n_, was a 82-D feature vector, where each element of this vector corresponds to a computed feature from the displacement of the COP, i.e., each subject has in total 82 computed feature from the displacement of the COP, considering the three COP directions (AP, ML and RD) and the two visual conditions (OE and CE).

In order to ease the data processing, the values of each feature of the feature vector were normalized between 0 and 1. An offset of 0.1 was added to the normalized vector for avoiding division by zero during the application of the signal processing stages.

Each feature from the pattern vector is represented by a Cartesian axis. Consequently, each subject will be represented by a point in this multidimensional space.

The next step of the signal processing is to reduce the multidimensional space into a one-dimensional space. The procedure of dimensional reduction consists of the rotation of an axis that is created imaginarily in the multidimensional space. With the rotation of the imaginary axis, became possible to verify in which position of this axis the projections of all points (i.e., all subjects) will provide the best discrimination of the seven groups.

It is important to note that an increase in the number of features to be analyzed increases the processing time significantly for each added feature, because of the amount of possible positions that may be assumed by the imaginary axis.

In this way, to process the data in a feasible time, a genetic algorithm was implemented to control the positions of the imaginary axis in the space. As the genetic algorithm [28,30] is a tool of fast search, it will find an angle of rotation optimized in a reduced time.

With the analysis of the projection of all the points on the imaginary axis, we can observe that the space, previously multidimensional, could be reduced for just one dimension, from where each subject will be represented by only one value (i.e., feature), that corresponds to their projection on the imaginary axis. Through this new value, so-called *LDA-value*, we can verify the degree of discrimination among the groups.

The quantification of discrimination between two groups is carried out through the accuracy estimator that consists in the relation between the average and the standard deviation of these two groups. The accuracy estimator *E *is given by (23), where  is the average of the *LDA-value *of group *x*,  is the average of *LDA-value *of group *y*, *σ*_*x *_is the standard deviation of *LDA-value *of group *x *and *σ*_*y *_is the standard deviation of *LDA-value *of group *y*.(23)

This estimator is an efficient statistical tool and it is responsible to indicate if one given feature is capable to discriminate the two groups in analysis, i.e., if there is a significant difference between them. The larger the value of *E*, the better will be the discrimination between the groups. If the estimator *E *results in a value larger than 1 (one), it can be concluded that the feature in analysis is capable of differentiating the groups.

To calculate the degree of discrimination among all groups, the value of *E *for each pair of existing groups was calculated, and then, the sum of these values resulted in a final value that characterizes the separation between all existing groups.

After the estimation of the best position of the imaginary axis, it was carried out the calculation of the relevance of each feature to the *LDA-value*. This calculation consists in the elimination of one feature to check the impact of it on the final discrimination of the groups. In such a way, the features that had an insignificant impact (i.e., relevance less than 1% of the accuracy estimator *E*) on the final discrimination of the groups were excluded from the analysis.

## Results

### Young group versus elderly group

Table 1 shows the definition of the young and elderly groups. Table 2 shows the mean and standard deviation of the values obtained for each feature for the young and elderly groups, with the opened eye (OE) and closed eye (CE) conditions. The value of probability (*p-value*) of the ANOVA test is only shown for the features that provided significant differences between the groups, and they are marked with an asterisk (*). The correlation of these features with the age of the subjects is shown through the *r *and *p-value*. The last column groups the features that had a Pearson's correlation coefficient larger than 0.9.

**Table 1:** Characterization of the subjects from the young and elderly groups.

	Young Adult (Groups 1 and 2)	Elderly Adult (Groups 6 and 7)
Age (years)	29.4 ± 4.93	77.83 ± 3.97

Age range (years)	21 to 39	73 to 87

Number of subjects	20	12

**Table 2:** Analysis of the computed features from the displacement of the COP.

	Young Adult (Groups 1 and 2)		Elderly Adult (Groups 6 and 7)		ANOVA *p-value*		Correlation with age		Correlation Groups
	
Features	OE	CE	OE	CE	OE	CE	OE	CE	
Mean Velocity	6.15	9.45	8.27	14.52	<		r = 0.3	r = 0.28	
Ap (mm/s) *	± 1.32	± 3.08	± 3.69	± 10.74	0.03				A

Mean Velocity	5.40	7.54	7.14	12.91	<	<	r = 0.25	r = 0.29	
Ml (mm/s) *	± 1.05	± 2.77	± 3.35	± 9.15	0.04	0.02			B

Mean Velocity	9.11	13.45	12.12	21.55	<	<	r = 0.29	r = 0.29	
RD (mm/s) *	± 1.59	± 4.05	± 5.43	± 15.63	0.03	0.04			C

Range	21.10	27.84	18.62	27.90			r = -0.03	r = -0.07	
Ap (mm)	± 5.57	± 8.03	± 4.74	± 10.80					D

Range	19.80	21.59	20.37	24.53			r = -0.05	r = 0.02	
Ml (mm)	± 6.40	± 7.32	± 7.98	± 10.43					E

Displacement	230.6	320.0	299.3	500.2	<	<	r = 0.27	r = 0.28	
Ap (mm) *	± 47.7	± 90.7	± 118.8	± 369.2	0.03	0.05			A

Displacement	171.4	235.8	229.4	414.7	<	<	r = 0.28	r = 0.3	
Ml (mm) *	± 30.8	± 81.6	± 102.1	± 300.6	0.03	0.02			B

Displacement	273.4	403.6	363.7	646.7	<	<	r = 0.29	r = 0.29	
RD (mm) *	± 47.6	± 121.4	± 163.0	± 469.2	0.03	0.04			C

RMS	4.52	5.74	3.60	4.91			r = 0.42	r = 0.48	
Ap (mm)	± 1.52	± 1.82	± 0.94	± 1.95			p < 0.01	p < 0.001	F

RMS	4.04	4.27	3.79	4.57			r = 0.33	r = 0.44	
Ml (mm)	± 1.39	± 1.47	± 1.22	± 1.82				p < 0.001	G

Mean Freq.	0.21	0.25	0.33	0.42	<	<	r = 0.41	r = 0.51	
Ap (Hz) *	± 0.06	± 0.07	± 0.16	± 0.19	0.01	0.001	p < 0.01	p < 0.001	F, H

Mean Freq.	0.22	0.28	0.30	0.41	<	<	r = 0.34	r = 0.46	
Ml (Hz) *	± 0.07	± 0.06	± 0.08	± 0.12	0.01	0.001	p < 0.01	p < 0.001	G, I

Peak Freq.	0.05	0.08	0.09	0.19			r = 0.27	r = 0.28	
Ap (Hz)	± 0.03	± 0.06	± 0.07	± 0.24					

Peak Freq.	0.06	0.10	0.07	0.15			r = 0.13	r = 0.25	
Ml (Hz)	± 0.03	± 0.07	± 0.05	± 0.09					

F50	0.083	0.13	0.20	0.27	<	<	r = 0.43	r = 0.48	
Ap (Hz) *	± 0.03	± 0.05	± 0.14	± 0.18	0.01	0.01	p < 0.001	p < 0.001	H

F50	0.11	0.16	0.17	0.28	<	<	r = 0.28	r = 0.45	
Ml (Hz) *	± 0.05	± 0.05	± 0.10	± 0.12	0.04	0.001		p < 0.001	I

F80	0.25	0.34	0.46	0.63	<	<	r = -0.29	r = -0.22	
Ap (Hz) *	± 0.11	± 0.12	± 0.26	± 0.33	0.01	0.001			D

F80	0.30	0.36	0.40	0.59	<	<	r = -0.14	r = -0.05	
Ml (Hz) *	± 0.11	± 0.08	± 0.14	± 0.23	0.03	0.001			E

Total Power	23.99	38.40	14.35	28.14			r = -0.28	r = -0.2	
Ap	± 17.88	± 25.04	± 7.44	± 23.95					D

Total Power	19.20	21.44	16.44	24.57			r = -0.16	r = -0.05	
Ml	± 12.80	± 14.53	± 10.54	± 19.45					E

Conf. Ellipse	223.0	325.5	172.6	305.3			r = -0.23	r = -0.14	
area (mm^2^)	± 128.9	± 210.4	± 93.0	± 249.5					J

Conf. Ellipse	10.77	12.29	8.79	11.02			r = -0.31	r = -0.2	
major axis (mm)	± 3.29	± 3.69	± 2.48	± 4.11					D, J

Conf. Ellipse	1.97	1.78	2.16	2.20		<	r = 0.09	r = 0.31	
angle (rad)*	± 0.38	± 0.36	± 0.58	± 0.40		0.01			

SDA - Ds	0.13	0.33	0.21	0.69			r = 0.21	r = 0.24	
Ap	± 0.06	± 0.21	± 0.18	± 0.89					A

SDA - Ds	0.12	0.23	0.21	0.58	<	<	r = 0.26	r = 0.3	
Ml *	± 0.04	± 0.16	± 0.17	± 0.57	0.04	0.01			B

SDA - Dl	0.05	0.05	0.02	0.02	<		r = -0.27	r = -0.23	
Ap *	± 0.04	± 0.05	± 0.02	± 0.04	0.04				

SDA - Dl	0.03	0.02	0.02	0.02			r = -0.16	r = -0.09	
Ml	± 0.02	± 0.03	± 0.02	± 0.02					

SDA - Hs	1.81	1.78	1.74	1.76	<		r = -0.31	r = -0.16	
Ap *	± 0.06	± 0.04	± 0.08	± 0.06	0.01				

SDA - Hs	1.80	1.77	1.77	1.78			r = -0.11	r = 0.03	
Ml	± 0.05	± 0.05	± 0.05	± 0.06					

SDA - Hl	0.64	0.44	0.32	0.23	<	<	r = -0.44	r = -0.44	
Ap *	± 0.22	± 0.19	± 0.24	± 0.17	0.001	0.01	p < 0.001	p < 0.001	

SDA - Hl	0.44	0.30	0.33	0.21			r = -0.23	r = -0.18	
Ml	± 0.20	± 0.13	± 0.17	± 0.13					

SDA-critical time	1.08	1.31	1.04	0.95		<	r = -0.1	r = -0.38	
Ap (s) *	± 0.51	± 0.31	± 0.41	± 0.42		0.01		p < 0.01	

SDA-critical time	1.43	1.28	1.25	0.98		<	r = -0.19	r = -0.38	
Ml (s) *	± 0.78	± 0.33	± 0.32	± 0.27		0.01		p < 0.01	

SDA (mm^2^)	0.16	0.41	0.17	0.43			r = -0.03	r = -0.02	
critical mag.-Ap	± 0.14	± 0.27	± 0.09	± 0.31					

SDA (mm^2^)	0.21	0.30	0.21	0.38			r = -0.08	r = 0.01	
critical mag.-Ml	± 0.18	± 0.22	± 0.13	± 0.27					

DFA - α	1.43	1.57	1.49	1.62			r = 0.16	r = 0.12	
Ap	± 0.09	± 0.09	± 0.13	± 0.16					

DFA - α	1.72	1.78	1.70	1.81			r = -0.09	r = 0.01	
Ml	± 0.06	± 0.09	± 0.12	± 0.12					

*H*_*R*/*S*_	0.97	1.01	0.99	1.02			r = 0.2	r = 0.04	
Ap	± 0.04	± 0.02	± 0.03	± 0.02					

*H*_*R*/*S*_	1.03	1.03	1.02	1.03			r = -0.09	r = -0.01	
Ml	± 0.004	± 0.005	± 0.01	± 0.01					

ApEn	0.008	0.008	0.010	0.012	<	<	r = 0.24	r = 0.43	
Ap *	± 0.002	± 0.002	± 0.003	± 0.005	0.05	0.01		p < 0.001	

ApEn	0.007	0.009	0.008	0.011		<	r = 0.14	r = 0.29	
Ml *	± 0.002	± 0.002	± 0.002	± 0.003		0.01			

Note that OE is the experimental condition of opened eyes whereas CE is the closed eyes. The mean and standard deviation are presented for each feature. Features that presented a significant difference between the groups are highlighted with '*' and in this case the estimated *p-value *of the ANOVA is shown. The correlation between the age and the feature is also presented. In the last column we present the analysis of the correlation between the studied features. When a correlation coefficient larger than 0.9 was found the features were included in the same group labeled by letters A, B, C, D, E, F, G, H, I, J.

From the analysis of Table 2 it is possible to conclude that the features that provided a significant difference between the two groups, for the OE and CE conditions, are: *mean velocity (ML and RD), total displacement (AP, ML *and *RD), mean frequency (AP *and *ML), F50 (AP *and *ML), F80 (AP *and *ML), Ds (ML), Hl (AP) *and *ApEn (AP)*. The values of *mean velocity (AP), Dl (AP) *and *Hs (AP) *provided only significant differences for the OE condition, whereas the values of *critical time (AP *and *ML), angle (RD) *and *ApEn (ML) *provided significant differences only for the CE condition.

Although some features (*mean frequency (AP *and *ML), F50 (AP *and *ML), Hl (AP), critical time (AP *and *ML) *and *ApEn (AP)*) had significant correlation with age, none had an r-value larger than 0.5, indicating that these features are weakly correlated with age. Furthermore, none of these features were able to discriminate the seven groups.

Moreover, we identified groups of features whose correlation estimated by the Pearson's correlation was larger than 0.9. Such features carry practically the same information, since they are highly correlated, and they are listed below:

• Group A: *mean velocity (AP), total displacement (AP), Ds (AP)*.

• Group B: *mean velocity (ML), total displacement (ML), Ds (ML)*.

• Group C: *mean velocity (RD), total displacement (RD)*.

• Group D: *range (AP), F80 (AP), total power (AP), major axis (RD)*.

• Group E: *range (ML), F80 (ML), total power (ML)*.

• Group F: *mean frequency (AP), RMS (AP)*.

• Group G: *mean frequency (ML), RMS (ML)*.

• Group H: *mean frequency (AP), F50 (AP)*.

• Group I: *mean frequency (ML), F50 (ML)*.

• Group J: *major axis (RD), area (RD)*.

### Results from the Linear Discrimination Analysis

As no computed feature from the displacement of the COP yielded a discrimination of the seven groups we applied the LDA for estimate of the *LDA-value*. For estimate of the *LDA-value *we took into account only the most relevant features as listed in Table 3. In total 35 features, that are presented in Table 3 were indentified. Note that each feature can be applied to the AP, ML or RD axis in two conditions, i.e., OE and CE. The letters in this table associate each relevant feature in the equations (24), (25) and (26), which estimate the *LDA-value*.(24)

**Table 3:** Features that have some influence on the *LDA-value*.

	AP axis		ML axis		RD axis	
**Measures**	**OE**	**CE**	**OE**	**CE**	**OE**	**CE**

*mean velocity*	(b)		(a)			

*total displacement*	(H)	(I)	(G)		(e)	

*range*	(d)		(c)			

*mean frequency*	(g)	(i)	(f)	(h)		

*peak frequency*	(k)		(j)	(l)		

*F80*			(m)	(n)		

*RMS*			(o)	(p)		

*Ellipse - major axis*					(q)	

*Ellipse - angle*					(r)	(s)

*SDA - Dl*	(t)	(u)				

*SDA - Hs*			(v)			

*SDA - Hl*	(x)					

*SDA - critical time*		(y)		(w)		

*SDA - critical magnitude*		(A)	(z)			

*DFA - α*				(B)		

*ApEn*		(E)	(C)	(D)		

*H*_*R*/*S*_		(F)				

All features that were considered relevant on the estimate of the *LDA-value*, according to the LDA method, are presented, and each letter of this table associate each relevant feature with the equations (24), (25) and (26) for estimating the *LDA-value*.

**Figure 1:**
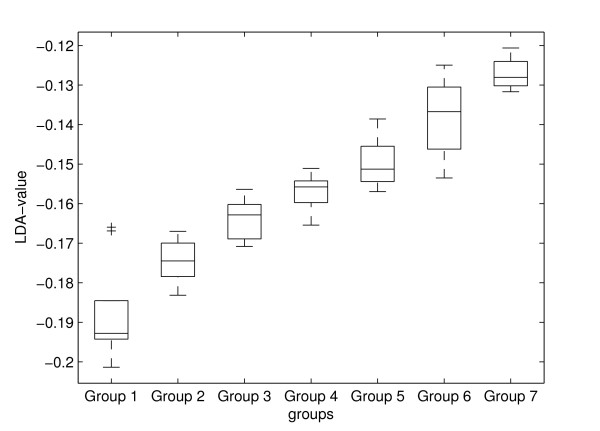
**Box plot of the *LDA-value *for the seven groups**. The graph shows the behavior of the *LDA-value *for the seven groups highlighting the differences among them.

Figure 1 depicts the results (box plot) obtained for the *LDA-value *for the seven groups. A visual inspection of the graph allows concluding that the *LDA-value *is a feature capable of discriminating the groups.

The ANOVA test was applied to these data. The results shown in Table 4 indicate that the *LDA-value *provided significant differences for all groups.

**Table 4:** *p-values *of ANOVA test for the *LDA-value *for the seven groups.

	Group 1	Group 2	Group 3	Group 4	Group 5	Group 6
Group 1	x					

Group 2	<0.01	x				

Group 3	< 10^-4^	<0.001	x			

Group 4	< 10^-5^	< 10^-5^	<0.01	x		

Group 5	< 10^-7^	< 10^-7^	< 10^-4^	<0.01	x	

Group 6	< 10^-7^	< 10^-7^	< 10^-5^	<0.001	<0.01	x

Group 7	< 10^-7^	< 10^-7^	< 10^-7^	< 10^-6^	< 10^-5^	<0.05

When estimating the correlation between the *LDA-value *and the age of the subjects, we obtained a Pearson's correlation coefficient equal to 0.914, indicating the high degree of correlation between the *LDA-value *and the age of the subjects.

**Figure 2:**
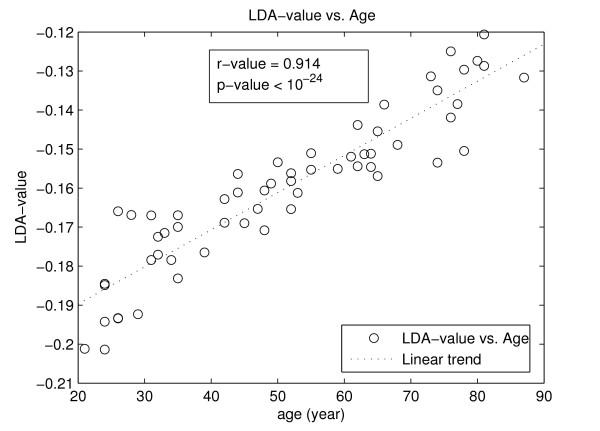
**Graph of age (years) vs. the *LDA-value***. The graph shows the linear trend of the *LDA-value *with age, highlighting the strong correlation between them as suggested by the *r *and *p-values*.

Figure 2 shows the graph between the age and *LDA-value *obtained for the subjects, where there is a linear trend between the two variables.

## Discussion

From Table 2 we can verify some features of the displacement of the COP that differentiate the young group from elderly group. These features show that the strategy of postural control, and consequently the displacement of the COP, changes between the groups. From the results we can note an increase in the *mean velocity *of the displacement of the COP in the elderly group, and also that the amplitude of the displacement of the COP remains constant (i.e., there is no changes in the values of *range*, *area of ellipse *and *RMS value*). This causes an increase in the total displacement and in the frequency of oscillation (*mean frequency*, *F50 *and *F80*) of the COP. Moreover, the displacement of the COP has a larger value of *ApEn *for the elderly group, showing that this signal becomes more unpredictable, having also a greater degree of randomness.

In general, the results obtained from Table 2 are in accordance with the results from other research groups [1,2,7,12,16,19,21,25,26], with a few exceptions. In our study, the coefficients of DFA (*α*) and Analysis R/S (H) did not allow a significant difference between the groups as shown in Table 2. This is not in accordance with the results obtained from Norris et al., 2005 [19]. Moreover, the values of the peak frequency, contrary to results published by McClenaghan et al., 1995 [14], did not result in a significant difference between the groups. However, the probability values (*p-value*) of the ANOVA test of these features were close to the acceptance threshold (*p-value *≈ 0.05). Therefore, it can be hypothesized that if the number of subjects increases, probably these features could yield a significant difference between the groups.

As in other studies [21,25], the results from Table 2 show that many traditional features seem to provide the same information, due to the high correlation between them, however, they differ in the fact that some are able to differentiate the young from elderly group and others not.

The DFA analysis did not provide significant differences between the two groups as shown in Table 2. However the value of *α *close to 1.5 characterized the displacement of the COP as a Brownian motion, or also known as random walk motion, which is in accordance with Collins and De Luca and other works [1,2,4-7,9,10,17,19,20,22].

Although Table 2 shows some features that are significantly different in the young and elderly groups, none of them provided significant difference among the seven groups and a strong correlation with age. Vieira TdMM et al., 2008 [26] found similar results, concluding that ageing itself does not result in significant changes of postural stability.

Contrary to many researches in the area [1,2,4-7,9,10,12-14,16,17,19-23,25,26], which only take into account two main groups (young and elderly) in their analysis, this study investigated seven distinct groups, allowing for a better characterization of changes that happen over the ageing. In addition, we noted from our results that commonly used features for COP analysis are not capable of providing clear correlations between these features and age for the seven studied groups. This was the main motivation for looking for alternative approaches, such as Linear Discriminant Analysis.

Through the Linear Discriminant Analysis, it was possible to combine the various features of the displacement of the COP in a unique feature, so-called *LDA-value*, whose value was able to separate and classify the seven groups, as can be observed in Figure 1. The *LDA-value*, as showed in Figure 2, has approximately a linear relationship with the age of the subjects, having a high Pearson's correlation coefficient (r = 0.914).

The results allowed us to verify that the LDA-value is a relevant feature for COP analysis, with potential application in a number of correlated studies in areas such as Physiotherapy, Neurology, Geriatrics and others. As the LDA-value showed to be linearly correlated with age for the group of healthy subjects, it is possible that this relation is not valid for patients with some disorders that deteriorate the postural control. In this case this parameter could potentially be employed for the diagnosis of some of these disorders. For instance, this parameter can be used for the characterization and monitoring of the progress of some neurological disorders that affect the postural control, such as peripheral vestibular syndromes.

Furthermore, the Linear Discriminant Analysis in this study can be extended by adding other features of the displacement of the COP that have not been used in this study, i.e., future studies may add other important features for the analysis of postural control, increasing the power of analysis of the *LDA-value*.

## Conclusion

This research showed a new method to analyze the postural control through the displacement of the COP. The *LDA-value *was effective in the discrimination of the seven groups, with a high degree of correlation with age of the subjects (r > 0.9).

As the *LDA-value *has a linear trend with the age of the subjects, it may have great importance in future researches. In this study we only considered the analysis of healthy subjects, but further investigations should be carried out in order to verify the behavior of the *LDA-value *in groups of subjects that had some disorders that deteriorate the postural control.

## Competing interests

The authors declare that they have no competing interests.

## Authors' contributions

All authors participated in the design of the study. GLC, MFSA and AOA participated in the data collection and in the development of the method procedure. GLC, AAP and AOA performed the data analysis and manuscript writing. All authors read and approved the final manuscript.
